# A 30-minute intravenous infusion of rituximab is safe and cost-effective (SpeedR)

**DOI:** 10.1007/s00277-025-06521-9

**Published:** 2025-07-31

**Authors:** Björn Engelbrekt Wahlin, Maria Ljungqvist, Ola Falk, Therese Rådman, Sandra Lindé, Kristina Sonnevi

**Affiliations:** 1 Division of Hematology, Department of Medicine, Huddinge, Karolinska Institutet, Stockholm, Sweden; 2 Medical Unit Hematology, Karolinska University Hospital, Solna, Cancer, Stockholm, Sweden

## Abstract

Rituximab is a monoclonal antibody used in the treatment of B-cell malignancies. Infusion reactions are common during the first exposure but decrease with subsequent infusions. We here present our experience with a protocol for a 30-minute intravenous infusion of rituximab (SpeedR) to patients who have previously tolerated a 90-minute rituximab infusion. All patients were premedicated with 10 mg cetirizine and 1 g paracetamol (steroids were only given when part of a concomitant chemotherapy regimen). In this cohort 268 patients received a total of 940 intravenous 30-minute infusions with only nine patients having adverse reactions (all grade 1–2) which required little or no treatment. Out of the nine patients with reactions, seven were planned for more rituximab treatment and all of them could later continue with 30-minute rituximab infusions. We conclude that 30-minute rituximab infusions are a safe and efficient way to optimize workflow in the outpatient clinic and to give cost-effective treatment to lymphoma patients.

## Introduction

Rituximab is a chimeric monoclonal antibody used in the treatment of B-cell non-Hodgkin lymphomas, chronic lymphocytic leukemia (CLL) and autoimmune diseases. When added to standard chemotherapy regimens, rituximab improved both progression-free and overall survival outcomes in patients with non-Hodgkin lymphoma and CLL [1]. Infusion-related reactions (IRR) are common during the first exposure but decrease with subsequent infusions. Therefore, the dose is titrated with a slow infusion rate at the first exposure to minimize the risk of infusion related reactions but in subsequent doses a higher infusion rate is applied.

Several studies have been conducted using rapid infusion protocols with rituximab given in 90–60 min from dose 2, if the previous dose was well-tolerated by the patient. In a 2010 review Atmar et al. concluded that after the first dose of rituximab has been given in a standard manner, subsequent doses can be safely administered by rapid-infusion protocols [2]. In recent years a subcutaneous preparation of rituximab has also become available and can be safely administrated in 5 min given that the patient previously received a full dose of rituximab intravenously [3]. Parallel to the introduction of the subcutaneous preparation, biosimilars of rituximab for intravenous use have also become available, at a substantially lower cost than the subcutaneous preparation.

## Methods

During the COVID-19 pandemic there was an urgent need to avoid crowding of patients at our outpatient clinic at the Hematology Unit, Karolinska Comprehensive Cancer Center. Furthermore, staff resources were limited and had to be used in a more effective way because many were sent to work in the COVID-19-care units of the hospital. We therefore introduced the SpeedR schedule: rapid intravenous infusion of 30 min of rituximab to all patients who had tolerated intravenous rituximab given in 90 min as the preceding dose (given within 3 months). Toleration of the previous dose was defined as it having been received without any dose interruption, infusion reactions, or need for emergency interventions.

SpeedR, 30-minute rituximab infusion, was introduced as a clinical pilot project in the outpatient clinic for lymphatic malignancies in November 2020; we have previously reported the safety data on the first 99 patients treated between November 2020 and December 2021 [4]. For this report we have reviewed data on patients who received SpeedR during 2023 and 2024 and combined them with the previous 99 patients, leading to a total number of 268 treated patients in this final analysis. Vital signs were recorded on a special form before start of SpeedR and after 30 min.

All patients receiving SpeedR had tolerated a previous dose of rituximab administered in 90 min and were premedicated at home with 1 g paracetamol and 10 mg cetirizine orally taken at least one hour before start of infusion. Prior steroids were only given when they were part of a concomitant chemotherapy regimen. Doses of rituximab were calculated according to indication with most patients receiving 375 mg/m^2^ and CLL patients receiving 500 mg/m^2^ intravenously with a steady infusion rate to finish at 30 min. The range of administered doses were between 500 mg to 1000 mg rituximab, diluted in 500 ml sodium chloride 0.9% in all cases. Blood pressure, temperature, oxygen saturation levels and pulse were measured before and immediately after the rituximab infusion and data were extracted from each patient’s file, including data on IRRs and their treatment. Patients who tolerated SpeedR continued with SpeedR in subsequent doses. Statistical analysis of the data was performed using STATA 14.2. The rituximab compound used was any approved biosimilar with the current best price on the market.

## Results

SpeedR was initiated 271 times in 268 patients (two patients with indolent B-cell lymphoma were treated twice with rituximab in different years, the second time at relapse; one was treated with single rituximab for marginal zone lymphoma in 2021 and with R-CHOP for transformation in 2024). The 268 patients received in total 940 doses of SpeedR; clinical features are presented in Table 1. Of the 116 aggressive malignancies, 15 were transformations from indolent B-cell lymphoma. In total, 17 patients had autoimmune hemolytic anemia or idiopathic thrombocytopenic purpura with or without malignant disease. Most patients (69%) started SpeedR as their 3rd dose of rituximab and 60% were 70 years or older.

**Table 1:** Clinical characteristics

Clinical feature	*N*		per cent	
Number of first SpeedR	271		100%	
Number of SpeedR doses given	940			
Sex				
Female	118		44%	
Male	153		56%	
Median age (years), range	74, 20–99			
20–60	65		24%	
60–69	42		15%	
70–79	87		32%	
80–99	77		28%	
Diagnosis				
* Aggressive malignancy*	116		43%	
Diffuse large B-cell lymphoma	111		41%	
Primary mediastinal B-cell lymphoma	2		1%	
Pre-B acute lymphoblastic leukemia	2		1%	
Follicular lymphoma grade 3B	1		0%	
* Indolent malignancy*	149		55%	
Follicular lymphoma grade 1–3 A	47		17%	
Marginal zone lymphoma	44		16%	
Mantle cell lymphoma	15		6%	
Indolent B-cell lymphoma	9		3%	
Waldenström’s macroglobulinemia	3		1%	
Nodular lymphocyte-predominant Hodgkin lymphoma	7		3%	
Chronic lymphocytic leukemia/small lymphocytic lymphoma	21		8%	
Hairy cell leukemia	3		1%	
* No malignancy*	6		2%	
Autoimmune hemolytic anemia	1		0%	
Acquired factor VIII deficiency	1		0%	
Idiopathic thrombocytopenic purpura	2		1%	
Monoclonal gammopathy of unknown significance	1		0%	
Thrombotic thrombocytopenic purpura	1		0%	
Rituximab only agent with SpeedR				
Yes	94		35%	
No	177		65%	
Concurrent agents				
CHOP or CHOP-like	124		46%	
GemOx	8		3%	
Bendamustine	36		13%	
Lenalidomide	5		2%	
Other chemo	4		1%	
Number of rituximab doses prior to first SpeedR in the current treatment line				
2	188		69%	
3	56		21%	
4	17		6%	
5 or more	10		4%	

There were no clinical differences in vital signs between measurements before and after the SpeedR infusion (Table 2). The number of patients with IRRs was low: eight common terminology criteria for adverse events (CTCAE) grade 1–2 during or after the first SpeedR and two after the second SpeedR in a total of nine patients (five women and four men; Table 3). No IRR grade 3–4 was recorded. Out of the nine patients with reactions, seven were planned for more rituximab treatment and all of them could later restart SpeedR, although one of these subsequently opted to continue with 90-minute infusions (Table 3). The rate of grade 1–2 reactions with first SpeedR was 8/271 (3.0%) and in all SpeedRs 10/940 (1.1%), [grade 2, 4/940 (0.4%)]. Only one patient among the 135 who had had steroids prior to their first SpeedR had a reaction, compared to seven among the 136 who did not; this was statistically significant (P [1-sided Fisher’s exact] = 0.036).

**Table 2:** Vital signs before and after first SpeedR

Vital signs with first SpeedR	Start	30 min
Median systolic blood pressure (mmHg), range	119, 83–178	120, 79–175
Median diastolic blood pressure (mmHg), range	71, 44–78	70, 41–78
Median pulse (beats per minute), range	70, 45–105	70, 42–106
Median temperature (Celsius), range	36.4°, 35.2°−37.5°	36.4°, 35.7°−37.1°
Median oxygen saturation level (per cent), range	98%, 93-100%	98%, 93-100%

**Table 3:** Adverse reactions

Patient identity	Diagnosis	Sex	Age (years)	No. prior rituximab	Concur-rent agents	Steroids	Reaction during/after first SpeedR	CTCAE grade	Action on next rituximab doses
SpeedR-42	Follicular lymphoma grade 2	F	64	2	Single	No	Felt queasy after 15 min infusion. Infusion was promptly paused for 10 min. Then the infusion was restarted at half speed.	1	Continued with SpeedR.
SpeedR-64	Marginal zone lymphoma	F	79	3	CHOP	Yes	Asymptomatic. BP went from 98/45 to 87/41 at stop. Received IV fluids, returned to 98/52.	2	Next rituximab infusion 90 min; subsequently SpeedR again.
SpeedR-67	Marginal zone lymphoma	M	53	3	Single	No	Asymptomatic. BP went from 109/67 to 87/61 at stop. Received IV fluids, returned to 99/65.	2	Next 2 rituximab infusions 90 min; subsequently SpeedR again.
SpeedR-164	Hairy cell leukemia	M	76	3	CdA	No	Chills. 100 mg hydrocortisone. Chills subsided.	2	None; this was the patient’s planned last dose of rituximab.
SpeedR-198	Chronic lymphocytic leukemia	M	77	3	Benda-mustine	No	2 h after stop: chills, hypertension, tachycardia. 100 mg hydrocortisone. Improved.	2	None; this was the patient’s last dose, because CR was attained.
SpeedR-200	Chronic lymphocytic leukemia	F	76	3	Benda-mustine	No	Reported having had chills at home in the evening after SpeedR.	1	Continued with SpeedR.
SpeedR-241	Marginal zone lymphoma	M	75	2	Single	No	Reported having had chills at home in the evening after SpeedR.	1	Continued with SpeedR.
SpeedR-268	Chronic lymphocytic leukemia with ITP	F	72	2	Single	No	After stop, patient had purple fingertips which normalized spontaneously within minutes.	1	Continued with SpeedR.
							*Reaction during/after second Speed R*		
SpeedR-203	Marginal zone lymphoma	M	80	2	Benda-mustine	No	Reported having had chills at home in the evening after the second SpeedR.	1	Continued with SpeedR.
SpeedR-200	Chronic lymphocytic leukemia	F	76	4	Benda-mustine	No	Reported having had chills at home in the evening also after the second SpeedR.	1	Continued with 90 min rituximab.

*ITP* idiopathic thrombocytopenic purpura, *F* female, *M* male, *BP* blood pressure, *IV* intravenous

We also looked at the use of resources in the clinic concerning patient-time in bed and time with nurses. The total patient-time in bed was reduced with 57% with SpeedR as compared to rituximab given in 90 min with a conservative estimate, calculated by the different total times of 45 min vs. 105 min (the extra 15 min includes infusion preparations, intravenous catheter establishment and intravenous catheter flush). SpeedR enabled an increase in the total number of outpatients in a bed per day with a factor of three if given as single treatment and a factor of two if given with chemotherapy. At the same time, the flexibility for nurses in the outpatient clinic was increased.

## Discussion

In this report we show that rituximab can be safely administered intravenously in 30 min to patients with CLL, B-cell lymphoma and/or autoimmune hemolysis/thrombocytopenia if they previously have tolerated a 90-minute infusion. This change in practice to 30-minute infusions has facilitated scheduling for nurses and allowed for higher throughput of patients in our outpatient ward.

It is since long known that the infusion time for rituximab can be shortened to 90 min from the second dose in patients who did not experience severe adverse events at dose 1 [5–7]. Furthermore, Dotson et al. have shown that 60-minute infusions to selected patients without steroid premedication are effective and safe [8]. Recently, a small Spanish prospective study of 32 doses of rituximab given in 30 min as second or third dose was well tolerated and safe [9].

30-minute rituximab infusions, which we called SpeedR, is today routinely given from the 3rd rituximab dose in our outpatient ward at Karolinska. The Swedish national archive of anticancer regimens has also incorporated SpeedR, a cost-effective way to treat patients with rituximab because biosimilars sell at a significantly lower cost than the subcutaneous preparation of MabThera^®^ but is almost as fast to give with our routine. Patients with subcutaneous treatment will occupy a bed at the clinic for only about 10 min less duration than patients receiving SpeedR, since controls of vital signs are indicated at least 15 min after the end of the subcutaneous injection. Hence, using the SpeedR protocol, we can increase the number of patients treated in a bed up to 300% without increasing the drug-related costs. SpeedR also increases the feasibility of fitting complicated multi-agent protocols into one workday without resorting to subcutaneous rituximab (such as R-polatuzumab vedotin-CHP).

Although it appears that prior steroids reduce the number of SpeedR reactions, we only use steroids routinely when they are part of the chemotherapy regimen. Steroids do have side effects of their own, and the adverse reactions were all grade 1–2 and could easily be handled with little or no treatment. SpeedR is given to all patients who tolerated a 90-minute infusion. This means that we do not perform any selection based on lymphocyte count or ECG before SpeedR, which makes the SpeedR protocol easy to apply in clinical routine.

We here show our experience from our routine treatment at a single centre, not from a clinical trial. There are no analyses with respect to long-term outcome; however, given the well-known pharmacokinetics and pharmacodynamics of rituximab, there is no reason to assume that SpeedR would reduce rituximab efficacy. We present a population-based, real-world cohort (the oldest patient was 99 years), with a wide range of diagnoses and 940 given SpeedR infusions. We find that there are very few adverse events and no serious ones.

We conclude that 30-minute infusions of rituximab are effective and safe. This is an efficient way to optimize workflow in the outpatient clinic and to give cost-effective treatment to lymphoma patients.

## Data Availability

No datasets were generated or analysed during the current study.
